# Opposing actions of CRF-R1 and CB1 receptor on facial stimulation-induced MLI-PC plasticity in mouse cerebellar cortex

**DOI:** 10.1186/s12868-022-00726-8

**Published:** 2022-06-26

**Authors:** Guang-Gao Li, Chun-Jian Piao, Peng Wan, Shu-Yu Li, Yu-Xuan Wei, Guo-Jun Zhao, Wen-Yuan Wu, Lan Hong, Chun-Ping Chu, De-Lai Qiu

**Affiliations:** 1 Department of Physiology and Pathophysiology, College of Medicine, Yanbian University, Yanji, 133002 Jilin China; 2 Department of Osteology, Affiliated Hospital of Yanbian University, Yanji, 133000 Jilin China; 3 Grade 2019 College Students Major in Clinical Medicine, College of Medicine, Yanbian University, Yanji, 133002 Jilin China; 4 Department of Physiology, College of Basic Medicine, Jilin Medical University, Jilin City, Jilin China; 5 Department of Urology, Affiliated Hospital of Yanbian University, Yanji, 133000 Jilin China

**Keywords:** Corticotropin-releasing factor (CRF), Mouse cerebellar cortex, Sensory stimulation, Molecular layer interneuron (MLI), Purkinje cell, * in vivo* cell-attached recording, Long-term plasticity

## Abstract

**Background:**

Corticotropin-releasing factor (CRF) is the major neuromodulator orchestrating the stress response, and is secreted by neurons in various regions of the brain. Cerebellar CRF is released by afferents from inferior olivary neurons and other brainstem nuclei in response to stressful challenges, and contributes to modulation of synaptic plasticity and motor learning behavior via its receptors. We recently found that CRF modulates facial stimulation-evoked molecular layer interneuron-Purkinje cell (MLI-PC) synaptic transmission via CRF type 1 receptor (CRF-R1)* in vivo* in mice, suggesting that CRF modulates sensory stimulation-evoked MLI-PC synaptic plasticity. However, the mechanism of how CRF modulates MLI-PC synaptic plasticity is unclear. We investigated the effect of CRF on facial stimulation-evoked MLI-PC long-term depression (LTD) in urethane-anesthetized mice by cell-attached recording technique and pharmacological methods.

**Results:**

Facial stimulation at 1 Hz induced LTD of MLI-PC synaptic transmission under control conditions, but not in the presence of CRF (100 nM). The CRF-abolished MLI-PC LTD was restored by application of a selective CRF-R1 antagonist, BMS-763,534 (200 nM), but it was not restored by application of a selective CRF-R2 antagonist, antisauvagine-30 (200 nM). Blocking cannabinoid type 1 (CB1) receptor abolished the facial stimulation-induced MLI-PC LTD, and revealed a CRF-triggered MLI-PC long-term potentiation (LTP) via CRF-R1. Notably, either inhibition of protein kinase C (PKC) with chelerythrine (5 µM) or depletion of intracellular Ca^2+^ with cyclopiazonic acid (100 µM), completely prevented CRF-triggered MLI-PC LTP in mouse cerebellar cortex * in vivo*.

**Conclusions:**

The present results indicated that CRF blocked sensory stimulation-induced opioid-dependent MLI-PC LTD by triggering MLI-PC LTP through CRF-R1/PKC and intracellular Ca^2+^ signaling pathway in mouse cerebellar cortex. These results suggest that activation of CRF-R1 opposes opioid-mediated cerebellar MLI-PC plasticity * in vivo* in mice.

**Supplementary Information:**

The online version contains supplementary material available at 10.1186/s12868-022-00726-8.

## Background

Corticotropin releasing factor (CRF) is a neuromodulator closely associated with stress responses that is synthesized and released in various neurons of the central nervous system, such as in the amygdala, thalamus, hypothalamus, hippocampus, and various brainstem nuclei [1–5]. Two types of CRF receptors are known, which are CRF-R1 and CRF-R2 [6]. Both CRF-R1 and CRF-R2 are expressed in the cerebellum of adult rodents [7–9]. CRF-R1 is found in all lobules of the cerebellar cortex, including Purkinje cells (PCs), molecular layer interneurons (MLIs), Golgi cells and granule cells [10–12]. In contrast, CRF-R2 has been found in the cerebellar cortical molecular layer, such as in parallel fibers and their terminals [11, 13]. CRF is released in cerebellar cortex by climbing fibers and CRFergic mossy fibers under physiological and stressful challenge conditions, which modulates neuronal circuitry function and motor learning behavior via its receptors [14–16].Cerebellar long-term synaptic plasticity is considered to be the cellular mechanism of motor learning [17, 18]. Numerous studies have demonstrated long-term plasticity at parallel fiber-PC (PF-PC), parallel fiber-MLIs (PF-MLIs), climbing fiber-PC and mossy fiber-granule cells under *in vitro* [19–25] and * in vivo* conditions [26–28]. In addition to excitatory synaptic plasticity, long-term synaptic plasticity at cerebellar MLI-PC inhibitory synapses, specifically long-term potentiation (LTP) and depression (LTD), has also been proposed to play critical roles for motor learning behavior [29, 30]. Under *in vitro* conditions, depolarization of PCs has been shown to induce LTP at MLI-PC synapses by an elevation of Ca^2+^ and retrograde activation of presynaptic N-methyl-d-aspartate (NMDA) receptors [29], as well as through an enhancement of postsynaptic GABAA receptor responsiveness [30]. In contrast, stimulation of PFs has been shown to induce LTD of MLI-PC synaptic transmission via activation of NMDA receptors in MLIs, which plays important roles during adaptation of horizontal optokinetic responses [31]. We previously found that 1 Hz stimulation of the ipsilateral whisker pad induces an opioid-dependent LTD of MLI-PC synaptic transmission via activation of NMDA receptors, suggesting that facial stimulation-evoked MLI-PC GABAergic synaptic plasticity plays a critical role in motor learning * in vivo* in mice [32].

CRF regulation of cerebellar circuitry synaptic transmission and plasticity has been well demonstrated [14, 33, 34]. In rat cerebellar slices, blockade of CRF receptors abolishes PF-PC LTD via a protein kinase C (PKC) signaling pathway, suggesting that CRF released from climbing fibers controls the induction of PF-PC LTD [14], whereas activation of CRF receptors induces climbing fiber-PC LTD through both PKC and PKA signaling pathways, suggesting that CRF regulates climbing fiber-PC synaptic plasticity [33]. Furthermore, CRF regulates cerebellar neuronal circuit function and motor behavior in response to stressful challenges [5, 35–37]. Activation of CRF-R1 plays an important role in responses to stressful challenges, and is critical in regulating particular forms of cerebellar learning [37, 38]. Moreover, activation of CRF-R1 increases excitability of MLIs and enhances facial stimulation-evoked MLI-PC synaptic transmission in mouse cerebellar cortex, suggesting that CRF modulates MLI-PC synaptic transmission and long-term plasticity * in vivo* in mice [12]. Although the mechanism of CRF system modulates neuronal circuitry function of cerebellar Purkinje cell and granule cell has been well investigated previously, the effect of CRF on facial stimulation-induced cerebellar MLI-PC synaptic plasticity is unknown. Therefore, we here investigated the effects of CRF on 1 Hz facial stimulation-induced opioid-mediated MLI-PC LTD in mouse cerebellar cortex by * in vivo* electrophysiological recording technique and pharmacological methods.

## Results

### Facial stimulation-induced MLI-PC LTD was blocked by CRF

**Fig. 1:**
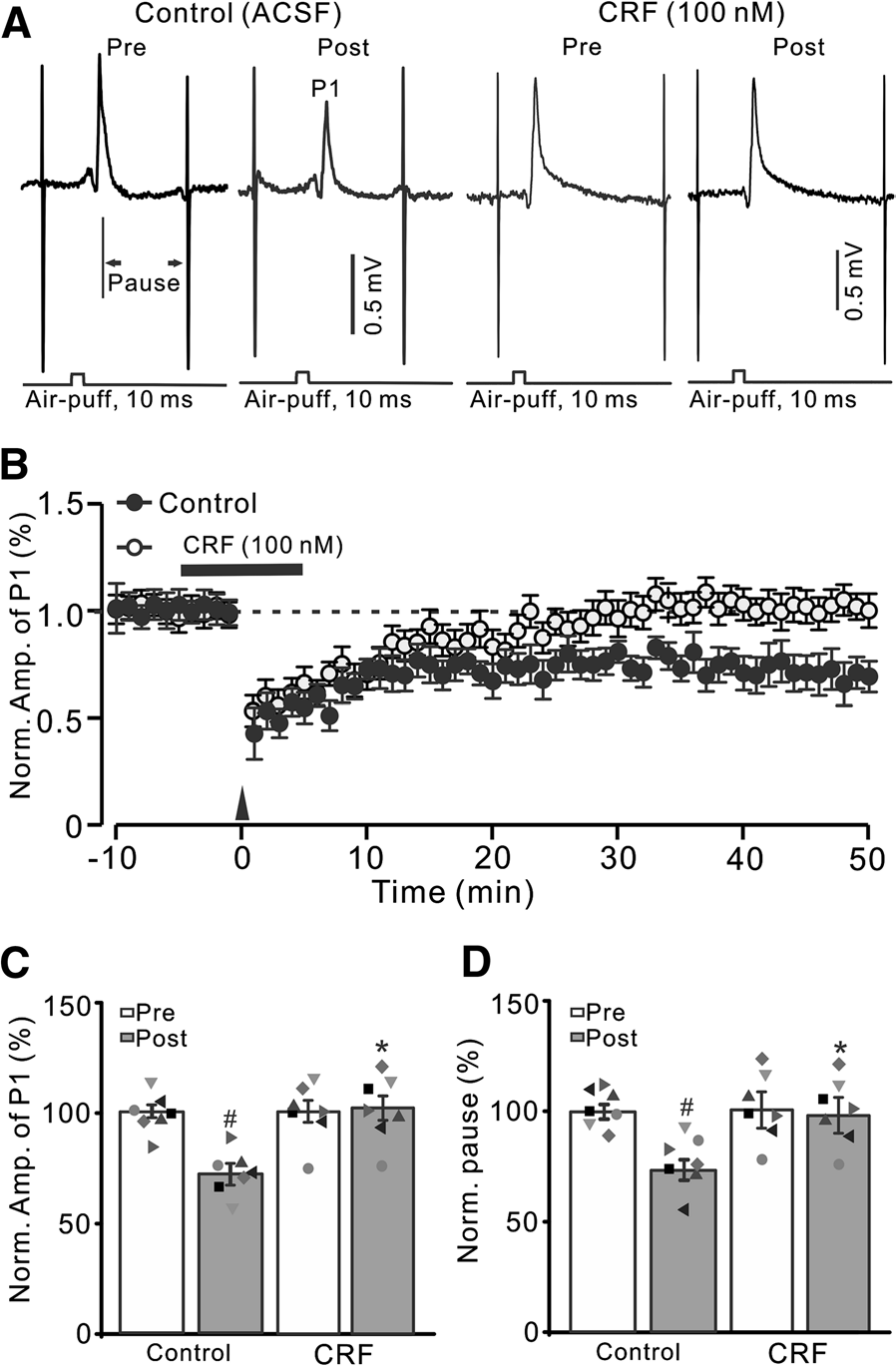
Effect of CRF on 1 Hz facial stimulation-evokedMLI-PC LTD *in vivo* in mice. **A** Upper: Representative cell-attached recording traces showing air-puff stimulation (10 ms, 60 psi; arrows)-evoked responses in cerebellar PCs before (Pre) and after (post) delivering 1 Hz (240 pulses) facial stimulation during treatments with ACSF (control) and CRF (100 nM). **B** Summary of data showing the time course of normalized P1 amplitude before and after delivery of 1 Hz facial stimulation (arrow head) in ACSF (control, filled circle) and CRF (open circles). **C** Bar graph with individual data showing the normalized amplitude of P1 before (Pre), after (Post) delivery of 1 Hz stimulation. **D** Mean (± S.E.M.) with individual data showing the normalized pause of simple spike firing before (Pre), after (Post) delivery of 1 Hz stimulation. ^#^P < 0.05 versus Pre of control; *P < 0.05 versus post of control. n = 7 mice in each group

Consistent with previous studies [32, 39], facial stimulation at 1 Hz (240 pulses) induced LTD at MLI-PC synapses (MLI-PC LTD). As shown in Fig. 1 Hz facial stimulation induced a persistent depression of MLI-PC GABAergic synaptic transmission, which was expressed as a decrease in amplitude of P1 under control conditions (ACSF; Fig. 1A, B). The normalized amplitude of P1 was decreased to 71.9 ± 4.6% of that of baseline during 40–50 min after 1 Hz facial stimulation (P < 0.05 versus baseline, n = 7 experiments; Fig. 1C), and the normalized pause of simple spikes was 73.3 ± 4.7% of that of baseline during 40–50 min after 1 Hz stimulation (P < 0.05 versus baseline, n = 7; Fig. 1D). Furthermore, 1 Hz facial stimulation delivered in the presence of CRF (100 nM) failed to induce MLI-PC LTD in (Fig. 1A, B). The normalized amplitude of P1 during 40–50 min after 1 Hz facial stimulation was 99.7 ± 4.2% of baseline (P > 0.05 versus baseline, n = 7; Fig. 1C), which was significantly higher than that of control (ACSF; P < 0.05, n = 7; Fig. 1C). The normalized pause time during 40–50 min after 1 Hz stimulation was 98.7 ± 4.3% of baseline (P > 0.05 versus baseline, n = 7; Fig. 1D), which was significantly different from that of control (ACSF; P < 0.05, n = 7; Fig. 1D). In addition, application of CRF (100 nM) application of CRF induced a transient slight increase, but did not induce a long-term change in amplitude of MLI-PC synaptic transmission (Additional file 1: Fig. S1). These results indicated that 1 Hz facial stimulation induced MLI-PC LTD under control conditions, but not in the presence of CRF. The results suggest that activation of CRF receptors prevents cerebellar MLI-PC LTD * in vivo* in mice.

### CRF prevented facial stimulation-induced MLI-PC LTD via CRF-R1

**Fig. 2:**
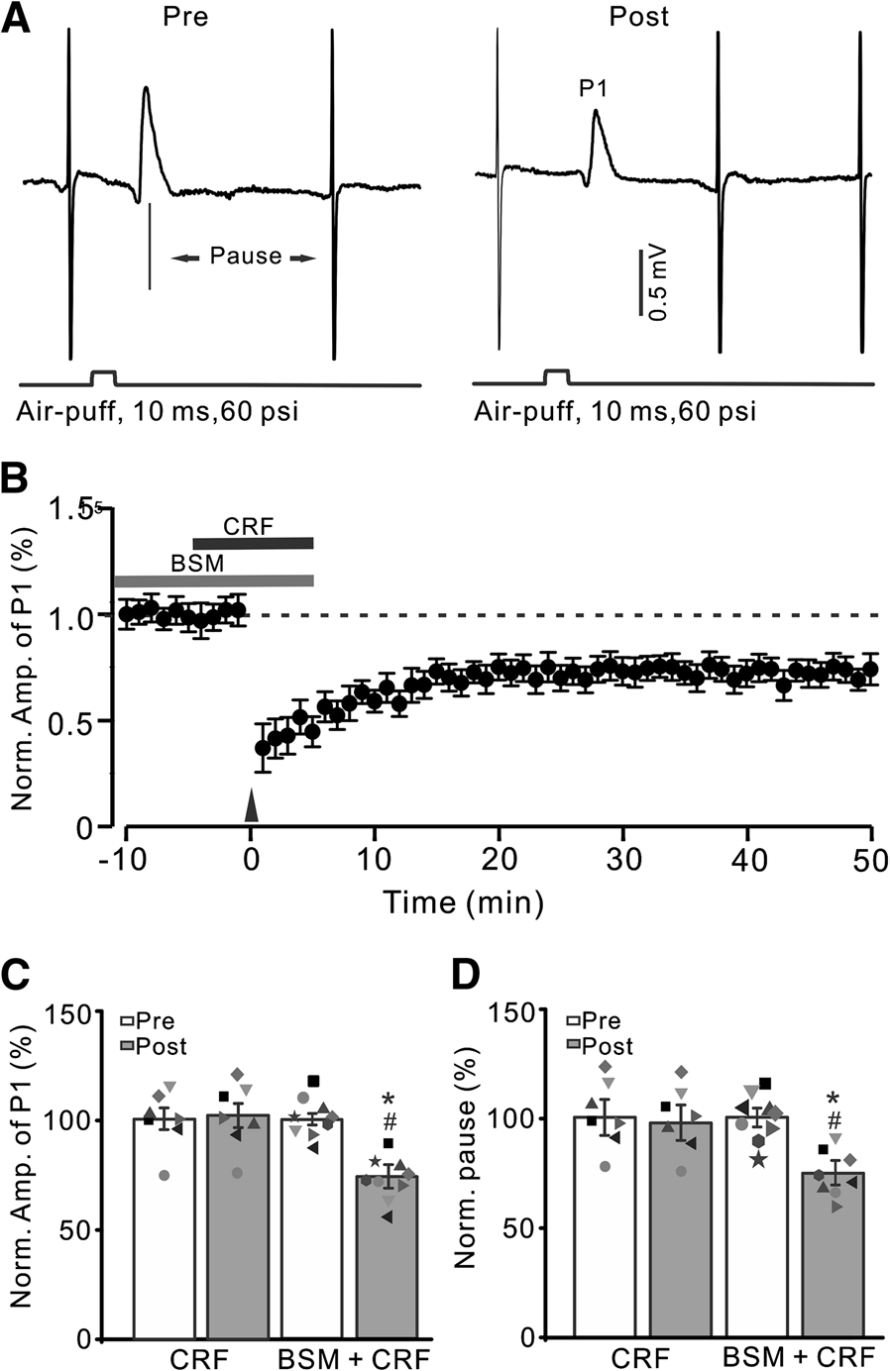
Blockade of CRF-R1, CRF failed to prevent facial stimulation-evoked MLI-PC LTD. **A** Upper: Representative cell-attached recording traces showing air-puff stimulation (10 ms, 60 psi; arrows)-evoked responses in a cerebellar PC before (Pre) and after (post) delivering 1 Hz (240 pulses) facial stimulation in the presence of a mixture of BSM (200 nM) and CRF (100 nM). **B** Summary of data showing the time course of normalized P1 amplitude before and after delivery of 1 Hz facial stimulation (arrow head) in the presence of the mixture. **C** Bar graph with individual data showing the normalized amplitude of P1 before (Pre), after (Post) delivery of 1 Hz stimulation. **D** Mean (± S.E.M.) with individual data showing the normalized pause of simple spike firing before (Pre), after (Post) delivery of 1 Hz stimulation. ^#^P < 0.05 versus Pre of BSM + CRF; *P < 0.05 versus post of CRF. n = 8 mice in each group

We used a selective CRF-R1 antagonist, BSM-763,534 (BMS, 200 nM) to examine whether CRF blocked MLI-PC LTD through CRF-R1. After blockade of CRF-R1, CRF failed to prevent the facial stimulation-induced MLI-PC LTD (Fig. 2A, B). The normalized amplitude of P1 was 72.4 ± 3.8% of baseline during 40–50 min after delivery of 1 Hz facial stimulation (P < 0.05 versus baseline, n = 7; Fig. 2C), which was significantly lower than that of CRF alone (P > 0.05, n = 7; Fig. 2C). The normalized pause time was 73.7 ± 4.2% of baseline during 40–50 min after delivery of 1 Hz facial stimulation (P < 0.05 versus baseline, n = 7; Fig. 2D), which was significantly shorter than that of CRF alone (P > 0.05, n = 7; Fig. 2D). These results indicated that in the absence of CRF-R1 activity, CRF failed to block the facial stimulation-induced MLI-PC LTD in mouse cerebellar cortex, suggesting that CRF inhibits facial stimulation-induced MLI-PC LTD via activation CRF-R1.

**Fig. 3:**
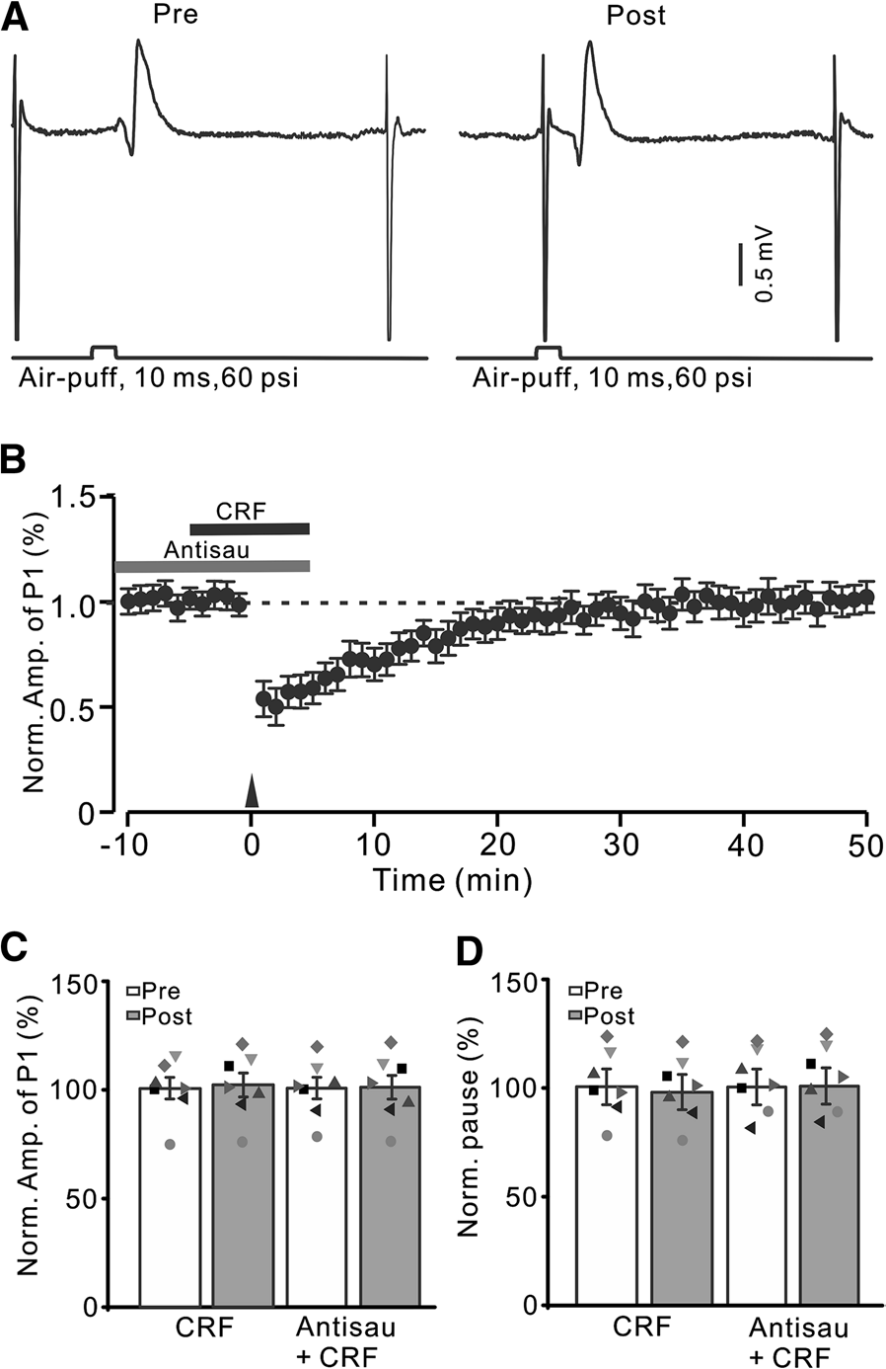
Blockade of CRF-R2 did not prevent the effect of CRF on facial stimulation-evoked MLI-PC LTD. **A** Upper: Representative cell-attached recording traces showing air-puff stimulation (10 ms, 60 psi; arrows)-evoked responses in a cerebellar PC before (Pre) and after (post) delivering 1 Hz (240 pulses) stimulation in the presence of a mixture of antisauvagine-30 (Antisau; 200 nM)and CRF (100 nM). **B** Summary of data showing the time course of normalized P1 amplitude before and after delivery of 1 Hz facial stimulation (arrow head) in the presence of Antisau and CRF. **C** Mean (± S.E.M) with individual data showing the normalized amplitude of P1 before (Pre), after (Post) delivery of 1 Hz stimulation. **D** Mean (± S.E.M.) with individual data showing the normalized pause of simple spike firing before (Pre), after (Post) delivery of 1 Hz stimulation. n = 7 mice in each group

We next used a selective CRF-R2 antagonist, antisauvagine-30 (200 nM) to examine whether CRF blocked MLI-PC LTD through CRF-R2. As shown in Fig. 3, blockade of CRF-R2 did not prevent the effect of CRF (100 nM) on facial stimulation-induced MLI-PC LTD (Fig. 3A, B). The normalized P1 amplitude was 100.1 ± 3.6% of baseline during 40–50 min after 1 Hz facial stimulation, (P > 0.05 versus baseline, n = 7; Fig. 3 C), which was similar to that of CRF alone (P > 0.05 versus CRF, n = 7; Fig. 3C). The normalized pause time was 99.4 ± 3.8% of baseline during 40–50 min after 1 Hz stimulation (P > 0.05 versus baseline, n = 7; Fig. 3D), which was not significantly different from that of CRF alone (P > 0.05 versus CRF, n = 7; Fig. 3D). These results indicated that CRF-R2 is not involved in effect of CRF on facial stimulation-induced MLI-PC LTD.

### Blockade of CB1 receptor-mediated MLI-PC LTD, CRF triggered MLI-PC LTP via CRF-R1

**Fig. 4:**
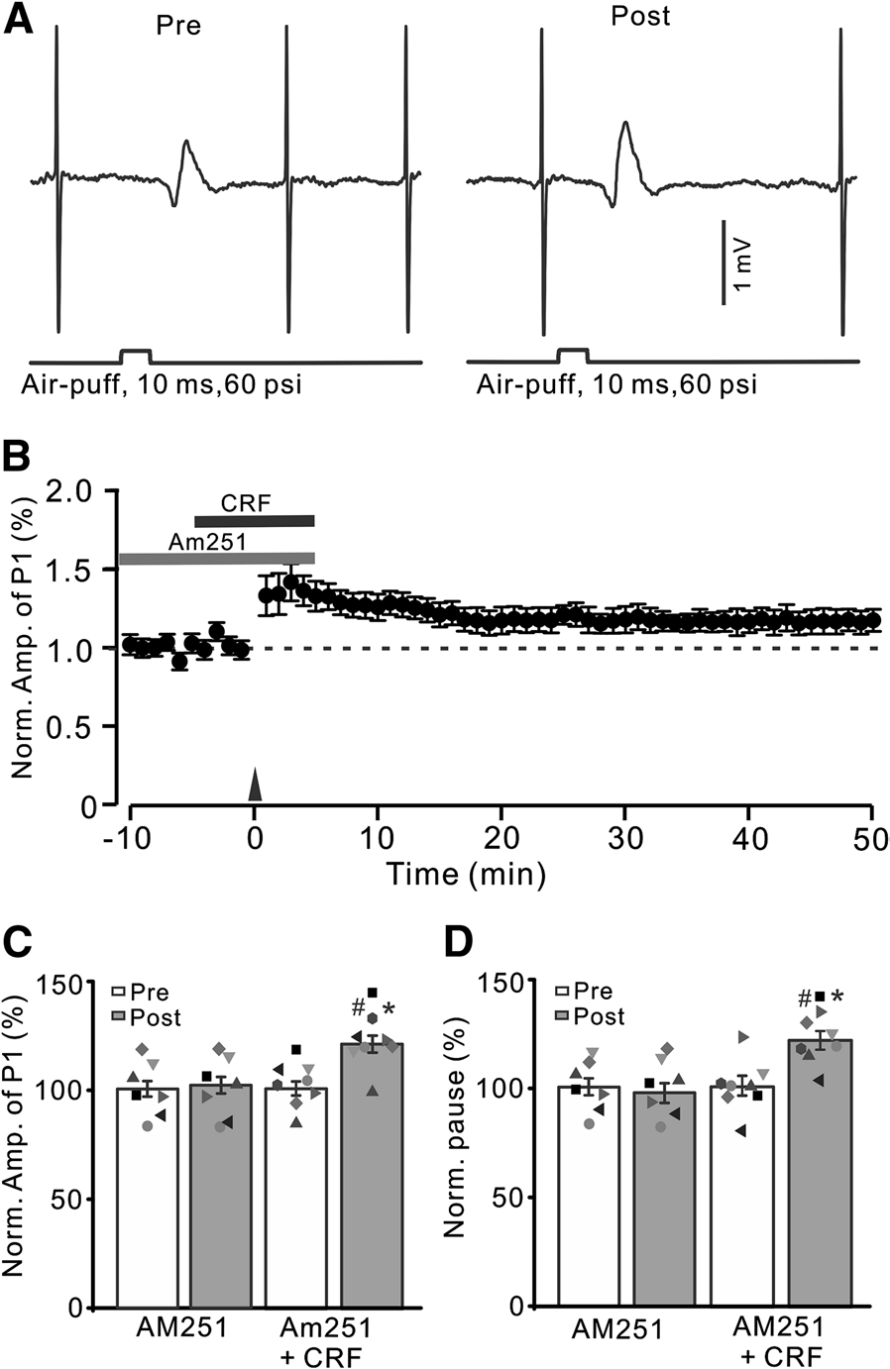
Blockade of CB1 receptor, CRF triggers MLI-PC LTP. **A** Upper: Representative cell-attached recording traces showing air-puff stimulation (10 ms, 60 psi; arrows)-evoked responses in a cerebellar PC before (Pre) and after (post) delivering 1 Hz (240 pulses) stimulation in the presence of a mixture of AM251(5 µM)and CRF (100 nM). **B** Summary of data showing the time course of normalized P1 amplitude before and after delivery of 1 Hz facial stimulation (arrow head) in the presence of AM-251 and CRF. **C** Bar graph with individual data showing the normalized amplitude of P1 before (Pre), after (Post) delivery of 1 Hz stimulation. **D** Mean (± S.E.M.) with individual data showing the normalized pause of simple spike firing before (Pre), after (Post) delivery of 1 Hz stimulation. ^#^P < 0.05 versus baseline (Pre) of AM251 + CRF; *P < 0.05 versus post of AM251. n = 8 mice in each group

Our previous study demonstrated that 1 Hz facial stimulation-induced MLI-PC LTD was dependent on CB1 receptors [32]. Consistent with the previous results (Bing et al., 2015), after blockade of CB1 receptors with AM-251 (5 µM), 1 Hz facial stimulation failed to induce MLI-PC LTD (Fig. 4C, D). However, after blockade of MLI-PC LTD with AM-251, facial stimulation triggered CRF to induce MLI-PC LTP (Fig. 4A, B). The normalized amplitude of P1 was 117.8 ± 5.9% of baseline during 40–50 min after 1 Hz facial stimulation (P < 0.05 versus baseline, n = 8; Fig. 4C), which was significantly higher than that of AM251 alone (P < 0.05 versus AM251, n = 8; Fig. 4C). The normalized pause time was 123.1 ± 7.6% of baseline during 40–50 min after 1 Hz stimulation (P > 0.05 versus baseline, n = 8; Fig. 4D), which was significantly higher than that of AM251 alone (P < 0.05 versus AM251, n = 8; Fig. 4D). These results indicated that after blockade of opioid-mediated MLI-PC LTD, 1 Hz facial stimulation triggered CRF to induce MLI-PC LTP in mouse cerebellar cortex.

**Fig. 5:**
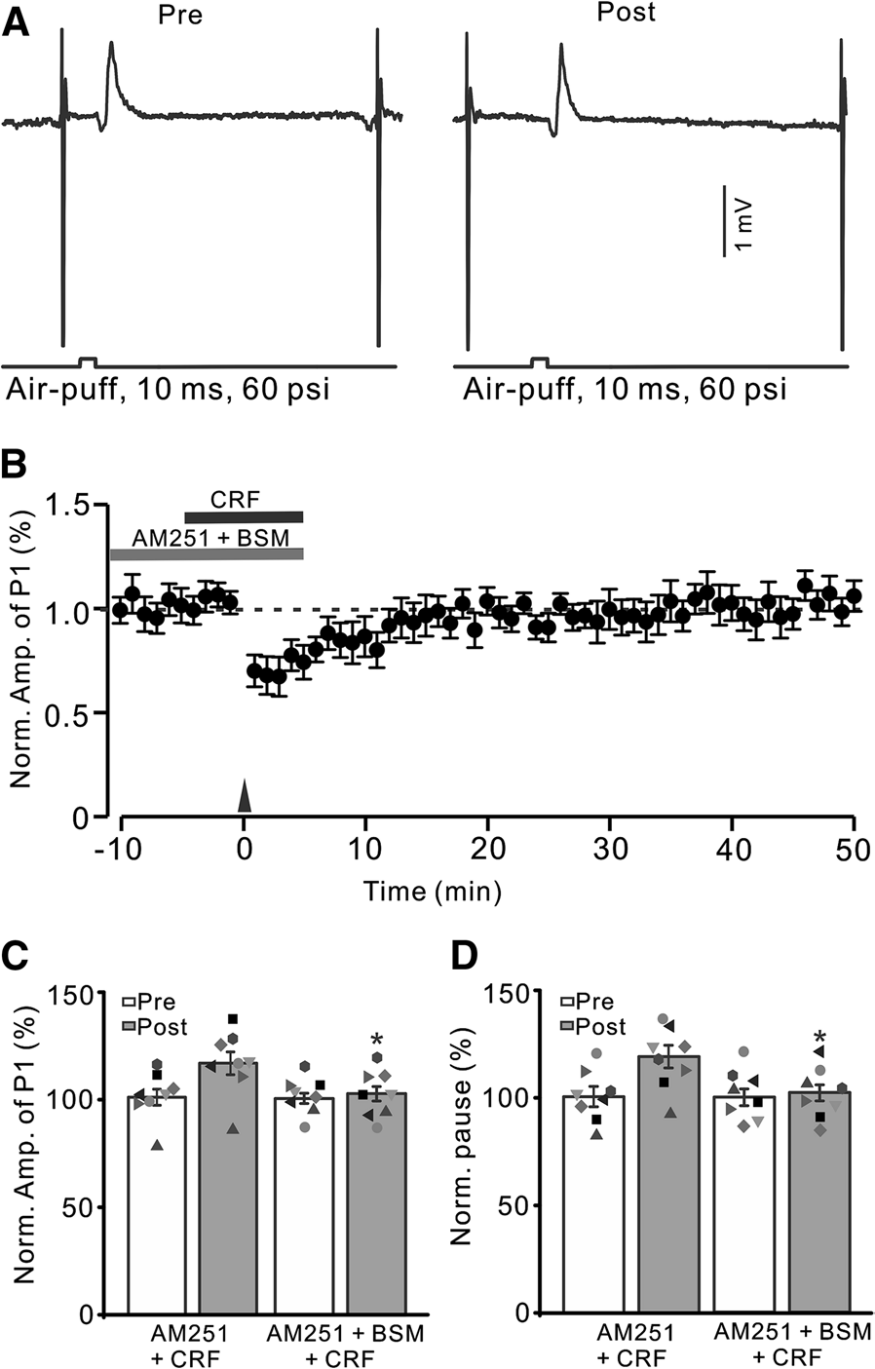
Blockade CB1 receptor and CRF-R1, CRF could not trigger MLI-PC LTP. **A** Upper: Representative cell-attached recording traces showing air-puff stimulation (10 ms, 60 psi; arrows)-evoked responses in a cerebellar PC before (Pre) and after (post) delivering 1 Hz (240 pulses) stimulation in the presence of a mixture of AM251(5 µM)+ BSM (200 nM) + CRF (100 nM). **B** Summary of data (n = 7) showing the time course of normalized P1 amplitude before and after delivery of 1 Hz facial stimulation (arrow head) in the presence of a mixture of AM251 + BSM + CRF. **C** Bar graph with individual data showing the normalized amplitude of P1 before (Pre), after (Post) delivery of 1 Hz stimulation. **D** Mean (± S.E.M.) with individual data showing the normalized pause of simple spike firing before (Pre), after (Post) delivery of 1 Hz stimulation. n = 8 mice in each group

We next examined whether CRF modulated 1 Hz sensory stimulation-induced MLI-PC LTP via CRF-R1. After blockade of CRF-R1, 1 Hz facial stimulation did not trigger CRF to induce MLI-PC LTP in mouse cerebellar cortex (Fig. 5). In the presence of a mixture of AM251 (5 µM), BSM (200 nM) and CRF (100 nM), delivery of 1 Hz facial stimulation did not induce MLI-PC plasticity (Fig. 5A, B). The normalized amplitude of P1 was 101.9 ± 3.4% of baseline during 40–50 min after 1 Hz facial stimulation (P < 0.05 versus baseline, n = 8; Fig. 5C), which was not significantly different from that of AM251 + CRF (P > 0.05 versus AM251 + CRF, n = 8; Fig. 5C). The normalized pause time was 102.2 ± 3.4% of baseline during 40–50 min after 1 Hz stimulation (P < 0.05 versus baseline, n = 8; Fig. 5D), which was not significantly different from that of AM251 + CRF (P > 0.05 versus AM251 + CRF, n = 8; Fig. 5D). The results indicated that upon blockade of CB1 receptor-mediated MLI-PC LTD, 1 Hz facial stimulation triggered CRF to induce MLI-PC LTP through CRF-R1.

### CRF modulated facial stimulation-induced MLI-PC LTP via PKC and intracellular Ca^2+^ signaling pathway

**Fig. 6:**
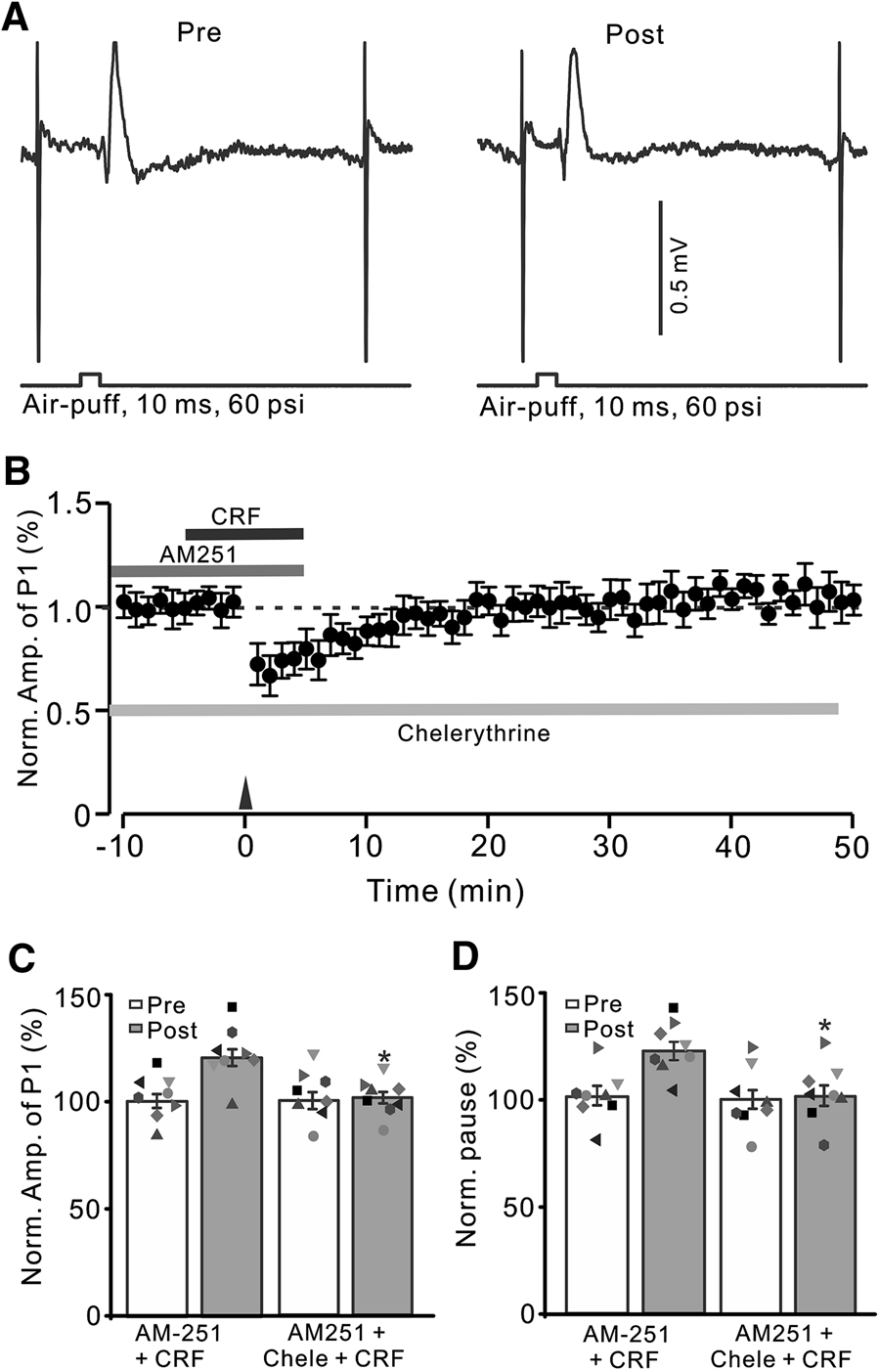
Blockade of PKC and CB1 receptor, CRF could not trigger MLI-PC LTP. **A** Upper: Representative cell-attached recording traces showing air-puff stimulation (10 ms, 60 psi; arrows)-evoked responses in a cerebellar PC before (Pre) and after (post) delivering 1 Hz (240 pulses) stimulation in the presence of a mixture of AM251 (5 µM) + chelerythrine (5 µM)+ CRF (100 nM). **B** Summary of data showing the time course of normalized P1 amplitude before and after delivery of 1 Hz facial stimulation (arrow head) in the presence of AM251 + chelerythrine + CRF. **C** Bar graph with individual data showing the normalized amplitude of P1 before (Pre), after (Post) delivery of 1 Hz stimulation. **D** Mean (± S.E.M.) with individual data showing the normalized pause of simple spike firing before (Pre), after (Post) delivery of 1 Hz stimulation. n = 8 mice in each group

It has been shown that activation of CRF-R1 modulates intracellular PKC and calcium levels, which regulates synaptic transmission and plasticity [40]. We next examined whether CRF induced MLI-PC LTP via the PKC signaling pathway by applying PKC inhibitor, chelerythrine [11, 41]. To fully inhibit the catalytic subunit of PKC, chelerythrine (5 µM) was perfused on the cerebellar surface over 30 min. After inhibition of PKC and CB1 receptors, 1 Hz facial stimulation failed to trigger CRF (100 nM) to induce MLI-PC LTP (Fig. 6A, B). In the presence of AM251 (5 µM), chelerythrine (5 µM) and CRF (100 nM), the normalized amplitude of P1 was 101.6 ± 3.7% of baseline during 40–50 min after 1 Hz facial stimulation (P < 0.05 versus baseline, n = 8; Fig. 6C), which was not significantly different from that of AM251 + CRF (P > 0.05 versus AM251 + CRF, n = 8; Fig. 6C). The normalized pause time was 100.6 ± 5.2% of baseline during 40–50 min after 1 Hz stimulation delivered in the presence of AM251 + CRF (P < 0.05 versus baseline, n = 8; Fig. 6D), which was not significantly different from that of AM251 + CRF (P > 0.05 versus AM251 + CRF, n = 8; Fig. 6D). The results indicated that upon inhibition of PKC, 1 Hz facial stimulation did not trigger CRF to induce MLI-PC LTP, suggesting that 1 Hz facial stimulation triggers CRF to induce MLI-PC LTP through the PKC signaling pathway.

**Fig. 7:**
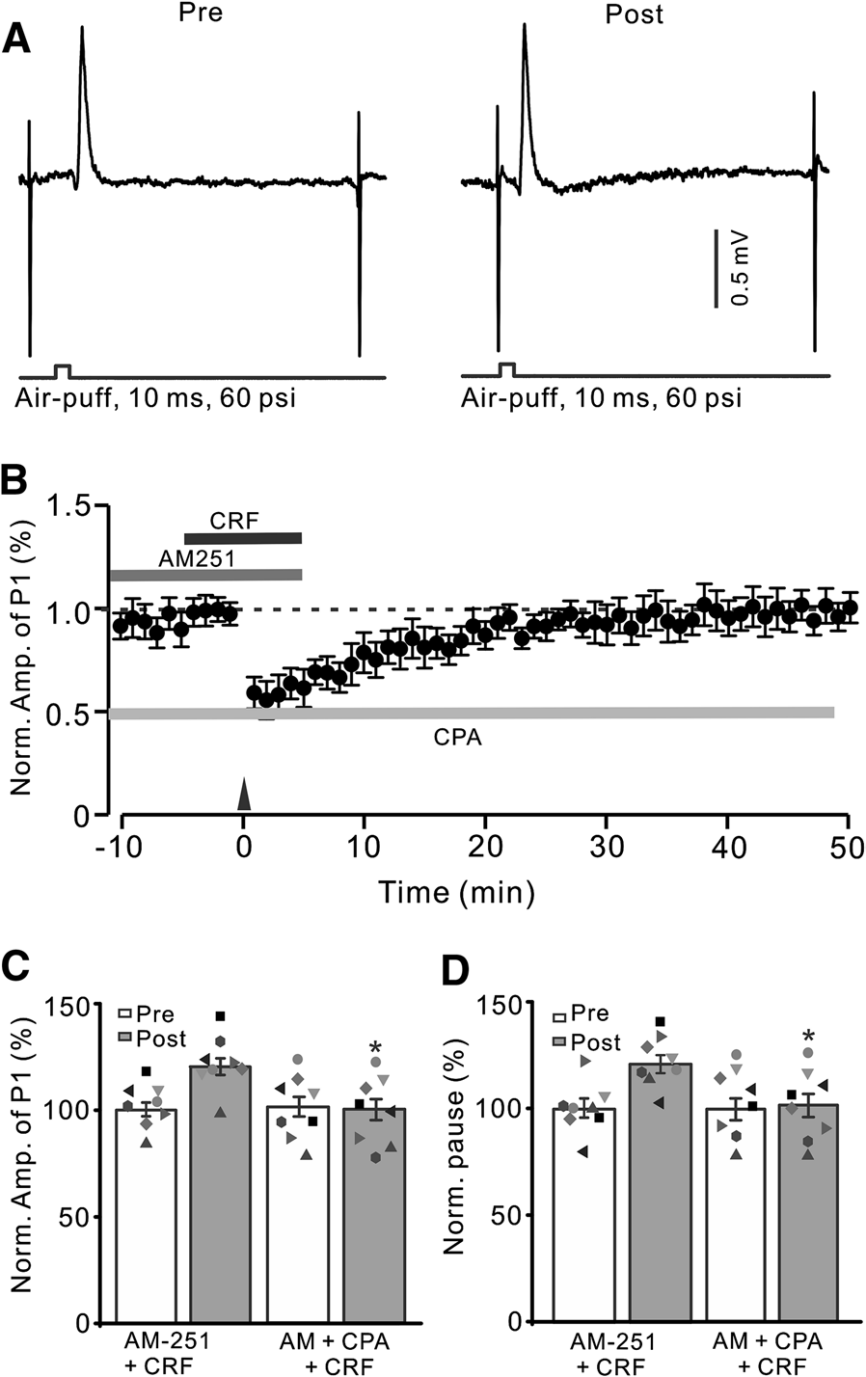
Depletion of intracellular Ca^2+^, CRF could not trigger MLI-PC LTP. **A** Upper: Representative cell-attached recording traces showing air-puff stimulation (10 ms, 60 psi; arrows)-evoked responses in a cerebellar PC before (Pre) and after (post) delivering 1 Hz (240 pulses) stimulation in the presence of a mixture of AM251 (5 µM) + cyclopiazonic acid (CPA, 100 µM) + CRF (100 nM). **B** Summary of data showing the time course of normalized P1 amplitude before and after delivery of 1 Hz facial stimulation (arrow head) in treatment with a mixture of AM251 + CPA + CRF. **C** Bar graph with individual data showing the normalized amplitude of P1 before (Pre), after (Post) delivery of 1 Hz stimulation. **D** Mean (± S.E.M.) with individual data showing the normalized pause of simple spike firing before (Pre), after (Post) delivery of 1 Hz stimulation. ^#^P < 0.05 vs. baseline (Pre) of AM-251 + CRF; *P < 0.05 vs. post of AM-251 + CRF. n = 8 mice in each group

Because CRF regulates transmitter release by mobilizing intracellular calcium release through a kinase-dependent pathway [42], we next examined whether 1 Hz facial stimulation triggered CRF to induce MLI-PC LTP when intracellular Ca^2+^ was depleted by perfusion of cyclopyruvate (CPA; 100 µM). To deplete intracellular Ca^2+^, CPA (100 µM) was perfused on the cerebellar surface over 30 min. As shown in Fig. 7, after depletion of intracellular Ca^2+^ and blockade of CB1 receptors, CRF (100 nM) failed to trigger 1 Hz facial stimulation-induced MLI-PC LTP (Fig. 7A, B). In the presence of CPA, AM251 and CRF, the normalized amplitude of P1 was 100.3 ± 4.3% of baseline during 40–50 min after 1 Hz facial stimulation (P < 0.05 versus baseline, n = 8; Fig. 7C), which was not significantly different from that of AM251 + CRF (P > 0.05 versus AM251 + CRF, n = 8; Fig. 7C). The normalized pause time was 99.8 ± 5.2% of baseline during 40–50 min after 1 Hz facial stimulation (P < 0.05 versus baseline, n = 8; Fig. 7D), which was not significantly different from that of AM251 + CRF (P > 0.05 versus AM251 + CRF, n = 8; Fig. 7D). The results indicated that upon depletion of intracellular Ca^2+^, 1 Hz facial stimulation did not trigger CRF to induce MLI-PC LTP, suggesting that the mechanism by which CRF induces MLI-PC LTP is dependent on intracellular Ca^2+^.

## Discussion

In this study, we found that CRF inhibited facial stimulation-evoked MLI-PC LTD via CRF-R1. Blockade of CB1 receptors abolished MLI-PC LTD, and revealed that facial stimulation triggered CRF to induce MLI-PC LTP via a CRF-R1/PKC/Ca^2+^ signaling pathway. These results indicate that activation of CRF-R1 triggers 1 Hz facial sensory stimulation-induced MLI-PC LTP through a PKC and intracellular Ca^2+^ signaling pathway, which opposes the opioid-mediated MLI-PC LTD * in vivo* in mice.

### Activation of CRF-R1 modulates cerebellar MLI-PC synaptic transmission and long-term plasticity

Electrophysiological recording from cerebellar PCs shows that sensory stimulation evokes MLI-PC GABAergic synaptic transmission, which expresses a strong inhibitory component * in vivo* in mice [32, 43, 44]. The sensory stimulation-evoked MLI-PC inhibitory synaptic transmission and plasticity play critical roles in regulating PC to output movement-related responses [32, 44]. Previous studies demonstrate that long-term synaptic plasticity at MLI-PC synapses can be induced by postsynaptic depolarization in cerebellar slices, and is dependent on activation of CB1 receptors [45, 46]. Under * in vivo* conditions, facial stimulation at 1 Hz induces an opioid-mediated MLI-PC LTD in mouse cerebellar cortex [32]. Consistent with our previous studies [32, 39], the present results showed that 1 Hz facial stimulation induced MLI-PC LTD, which was abolished by a CB1 receptor antagonist, indicating that MLI-PC LTD is opioid-dependent. Interestingly, delivery of 1 Hz facial stimulation in the presence of CRF failed to induce MLI-PC LTD, indicating that activation of CRF receptors inhibits the induction of MLI-PC LTD.

Immunohistochemical study has indicated that both CRF-R1 and CRF-R2 are expressed in adult rat cerebellum [47]. Activation of cerebellar afferents induces CRF release from terminals of climbing fibers and some mossy fibers in all lobules of rat cerebellum [4]. CRF release from mossy fiber terminals in the granular layer regulates mossy fiber-granule cell synaptic function, and CRF release from climbing fiber terminals in the molecular layer modulates PF-PC, PF-MLIs and MLI-PC synaptic transmission and plasticity [11, 12, 48, 49]. Notably, CRF-R1 is present on the somas and axonal terminals of MLIs, suggesting that CRF release from climbing fiber terminals modulates the activity of MLIs and their GABAergic synaptic transmission via CRF-R1 [48]. We recently found that CRF increased excitability of MLIs, resulting in a significant enhancement of facial stimulation-evoked MLI-PC synaptic transmission via CRF-R1 * in vivo* in mice, indicating that CRF modulates MLI-PC synaptic transmission and long-term plasticity and plays a critical role in motor learning in living animals [12]. The present results show that CRF fails to prevent the facial stimulation-induced MLI-PC LTD in mouse cerebellar cortex in the presence of a CRF-R1 antagonist, indicating that activation of CRF-R1 inhibited the expression of MLI-PC LTD. However, CRF blocked facial stimulation-induced MLI-PC LTD in the presence of a CRF-R2 antagonist, suggesting that CRF inhibits facial stimulation-induced MLI-PC LTD via CRF-R1 rather than CRF-R2. These results are consistent with previous studies [12, 48, 49], indicating that CRF-R1 is expressed in MLIs and contributes to the modulation of MLI-PC synaptic transmission and long-term synaptic plasticity in cerebellar cortex.

### Pharmacological blockade of MLI-PC LTD, facial stimulation triggers CRF to induce MLI-PC LTP through CRF-R1

CB1 receptor-mediated LTD has been demonstrated in several brain areas at both inhibitory and excitatory synapses [50]. In cerebellar cortex, opioid-dependent PF-PC LTD has been demonstrated in cerebellar slices [24], and in living animals [28]. A presynaptically-expressed form of opioid-dependent LTD has been also found at PF-MLI synapses [51]. Our previous study demonstrated that 1 Hz facial stimulation-induced MLI-PC LTD was blocked by a CB1 receptor antagonist, indicating that facial stimulation-induced MLI-PC LTD is opioid-dependent [32]. The present results show that 1 Hz facial stimulation triggers CRF to induce MLI-PC LTP in the presence of a CB1 receptor antagonist, indicating that blockade of opioid-dependent LTD revealed CRF-triggered MLI-PC LTP in mouse cerebellar cortex. In the presence of a CRF-R1 antagonist, CRF did not induce MLI-PC LTP in mouse cerebellar cortex. These results indicate that upon blockade of opioid-mediated MLI-PC LTD, facial stimulation activates CRF-R1 to induce MLI-PC LTP * in vivo* in mice. In addition, the amplitude of the facial stimulation-induced MLI-PC LTP was approximately 17% of baseline, which is lower than the amplitude of PF-PC LTP induced by 4 Hz stimulation in cerebellar slices [23]. The low amplitude of the CRF-R1-triggered MLI-PC LTP might be intrinsic, but does not seem to be caused by anesthesia. Although most anesthetics, such as propofol, etomidate and ketamine, enhance GABA_A_ receptor activity or GABAergic synaptic transmission [52–54], urethane depresses neuronal excitability through activation of barium-sensitive potassium leak conductance, without effects on excitatory glutamate- or inhibitory GABA-mediated synaptic transmission [55]. Therefore, anesthesia with urethane has less effect on facial stimulation-evoked MLI-PC synaptic transmission and long-term synaptic plasticity * in vivo* in mice [32, 39, 43, 44].

### **Blockade of MLI-PC LTD, facial stimulation triggers CRF to induce MLI-PC LTP via PKC/Ca**^**2+**^**signaling pathway**

CRF-R1 is G-protein coupled receptor that can activate adenylate cyclase, PKA, PKC and intracellular second messenger cyclic adenosine monophosphate, thereby increasing the level of intracellular Ca^2+^ [56–58]. Previous studies have demonstrated that activation of CRF-R1 enhances GABA release via the PKC signaling pathway [56–58]. CRF modulates GABAergic synaptic transmission of serotonergic neurons in pyramidal neurons of the prefrontal cortex [58], and enhances GABA release in the central amygdala through a CRF-R1/PKC signaling pathway [57]. Although PKC signaling cascade-dependent LTP has not been demonstrated in cerebellar cortex, PKC activation has been found to be required for LTP induction in other areas of the brain [59, 60]. Consistent with previous studies [Luu and Malenka 59, MacDonald et al. 60], the present results showed that inhibition of PKC prevented CRF from inducing MLI-PC LTP, suggesting that 1 Hz facial stimulation triggers CRF to induce MLI-PC LTP through the PKC signaling pathway. In addition, activation of CRF-R1 regulates transmitter release by a kinase-dependent mobilization of calcium release from intracellular stores [40], and CRF-activated presynaptic intracellular calcium stores might serve as reservoirs for release machinery [61]. The present results showed that after depletion of intracellular Ca^2+^, 1 Hz facial stimulation did not trigger CRF to induce MLI-PC LTP, suggesting that 1 Hz facial stimulation-induced CRF-triggered MLI-PC LTP requires CRF-R1 and intracellular Ca^2+^. CRF may activate CRF-R1 in somas and axonal terminals of MLIs to induce presynaptic MLI-PC LTP via a PKC/Ca^2+^ signaling pathway [4, 48]. Alternatively, CRF may activate CRF-R1 in somas and primary dendrites of PCs, which may contribute to the induction of MLI-PC LTP via a postsynaptic PKC/Ca^2+^ signaling pathway. To clarify whether CRF induces MLI-PC LTP through presynaptic and/or postsynaptic CRF-R1, more experiments are required using living animals in the future.

CRF, also called the stress hormone, is the major neuromodulator during the stress response. CRFergic neurons are found in the amygdala, thalamus, hypothalamus, and various brain stem nuclei [3, 35]. CRFergic mossy fibers from such brain regions project into the cerebellum, suggesting that CRF is involved in the regulation of cerebellar circuit function and motor behavior in response to stressful challenges [5]. Furthermore, release of CRF from climbing fiber terminals under physiological and challenge conditions may contribute to modulation of cerebellar parallel fiber LTD and motor learning behavior [14–16]. Under physiological conditions, release of CRF from climbing fiber terminals controls cerebellar LTD via a PKC signaling pathway, indicating that CRF modulates motor learning behavior [14]. Reduction of CRF level in the inferior olivary nucleus induces motor deficiency under stressful challenges, regardless of basal locomotion or anxiety-like behavior, and stressful stimulation upregulates CRF mRNA level for a complex motor response [37]. Therefore, it is reasonable to hypothesize that release of CRF during stressful challenges and classical conditioning behaviors contributes to MLI-PC plasticity and motor learning [16, 17]. Moreover, 1 Hz facial stimulation induces endocannabinoids release from MLIs via activation of NMDA receptors, which produces CB1 receptor-dependent MLI-PC LTD in mouse cerebellar cortex * in vivo* [32]. Accordingly, our present results showed that CRF inhibited opioid-induced MLI-PC LTD via the CRF-R1/PKC signaling pathway, which suggests that CRF may impair motor learning behavior through opposing the opioid-mediated MLI-PC LTD * in vivo* in mice. In addition, cerebellar LTD has been proposed to provide a cellular mechanism for motor learning [17], thus the facial stimulation-induced MLI-PC LTD might be a form of motor learning behavior [32], and blockade of MLI-PC LTD by CRF might be related to disrupt motor learning behavioral procedure in mice [5, 38]. Since CRF modulates neuronal microcircuit function of cerebellum, it is reasonable to consider that motor learning may be modified by CRF via CRF-R1 during stressful challenge behaviors [5]. The present results provide valuable data for further understand the relationship between CRF signaling and opioid modulation during the sensory stimulation-induced synaptic plasticity at MLI-PC synapses. Taken together, our present results indicate that after blockade of CB1 receptor-dependent MLI-PC LTD, 1 Hz facial stimulation triggers CRF to induce MLI-PC LTP through the CRF-R1/PKC/Ca^2+^ signaling pathway. Our experimental paradigms may allow us to unravel a possible mechanism of stressful challenges modulate motor learning behavioral procedure * in vivo* in mice.

## Conclusions

The present results indicate that CRF blocks sensory stimulation-induced opioid-dependent MLI-PC LTD, by triggering MLI-PC LTP through CRF-R1/PKC and intracellular Ca^2+^ signaling pathway, suggesting that activation of CRF-R1 plays an opposing action on opioid-mediated cerebellar MLI-PC plasticity * in vivo* in mice. The present study provides novel evidence for better understanding the mechanisms of CRF modulation of sensory stimulation-induced cerebellar MLI-PC synaptic plasticity and its implications for motor learning in rodents.

## Materials and methods

The procedures of anesthesia and surgical have been described previously. The experimental procedures were approved by the Animal Care and Use Committee of Yanbian University. The permit number is SYXK (Ji) 2011-006. All the experimental methods were in accordance with the animal welfare guidelines of the U.S. National Institutes of Health, and the Animal Research: Reporting * in vivo* Experiments (ARRIVE; https://arriveguidelines.org). Male adult (6-8-week-old; n = 92) C57BL/6 mice were bought from the experiment center of Yanbian University. All mice were housed under a 12-h light: 12-h dark cycle with free access to food and water in a colony room under room temperature (23 ± 1 °C) and humidity (50 ± 5%). The anesthesia and surgical procedures have been described previously (Chu et al. [44]). Since urethane anesthesia has a stable anesthetic state and does not impair excitatory glutamatergic and inhibitory GABAergic synaptic transmission, we used urethane to anesthetize animals [32, 39, 41, 42, 55]. After the mice were anesthetized with urethane (1.3 g/kg body weight i.p.), and were tracheotomized to avoid respiratory obstruction. After the mice were fixed on a custom-made stereotaxic frame, soft tissue was stripped to gain access to the dorsal portion of the occipital bone. A watertight recording chamber was created and a 1–1.5 mm craniotomy was drilled to expose the cerebellar surface corresponding to Crus II. The brain surface was constantly perfusion with oxygenated artificial cerebrospinal fluid (ACSF: 125 mM NaCl, 3 mM KCl, 1 mM MgSO4, 2 mM CaCl_2_, 1 mM NaH_2_PO_4_, 25 mM NaHCO_3_, and 10 mM D-glucose) with a peristaltic pump (Gilson Minipulse 3; Villiers, Le Bel, France) at 0.5 ml/min. Rectal temperature was monitored and maintained at 37.0 ± 0.2 °C using body temperature equipment.

An Axopatch-200B amplifier (Molecular Devices, Foster City, CA) was employed to perform cell-attached recordings from PCs. The signal of PC activity was acquired through a Digidata 1440 series analog-to-digital interface on a personal computer using Clampex 10.3 software. Patch pipettes were prepared with a puller (PB-10; Narishige, Tokyo, Japan) from thick-wall borosilicate glass (GD-1.5; Narishige), with resistances of 3–5 MΩ. The cell-attached recordings from PCs were performed at depths of 200–250 μm under the pia mater membrane, and were identified by their simple spikes (SS) and complex spikes (CSs).

Facial stimulation was performed by air-puff (10 ms, 60 psi) of the ipsilateral whisker pad through a 12-gauge stainless steel tube connected with a pressurized injection system (Picospritzer^®^ III; Parker Hannifin Co., Pine Brook, NJ, USA). The air-puff stimulation (duration: 25 s; intersweep interval: 30 s) were synchronized with the electrophysiological recordings and delivered at 0.05 Hz via a Master 8 controller (A.M.P.I., Jerusalem, Israel) and Clampex 10.3 software. Under cell-attached recordings conditions, air-puff stimulation of ipsilateral whisker pad (10 ms, 60 psi) induced a large positive component (P1) followed by a pause of simple spike firing (Fig. 1A). Application of GABA_A_ receptor antagonist, GABAzine (20 µM) abolished P1 and revealed the facial stimulation-evoked simple spike firing (Additional file 2: Fig. S2). According to our previous studies [32, 44], the facial stimulation-evoked P1 was identified as MLI–PC GABAergic synaptic transmission. Measurement of amplitude of P1 and the pause time of simple spike firing could reflect the strength of MLI-PC GABAergic synaptic transmission [32, 39]. Because MLI-PC LTD could be induced by 1 Hz, but not 2 and 4 Hz facial stimulation [32], we used 1 Hz (240 pulses) air-puff stimulation (10 ms, 60 psi) to induce MLI-PC LTD.

The reagents included human/rat CRF (Peptide Institute Inc., Japan); N-(piperidin-1-yl)-5-(4-iodophenyl)-1-(2,4-di-chlorophenyl)-4-methyl-1 H-pyrazole-3-carboxamide (AM251), CB1 receptors blocker; chelerythrine, PKC inhibitor; cyclopiazonic acid (CPA), intracellular Ca^2+^ depletion; BMS-763,534, CRF-R1 antagonist, and antisauvagine-30, CRF-R2 antagonist were purchased from Sigma-Aldrich (Shanghai, China). The stock solutions of BMS-763,534 were dissolved in dimethyl sulfoxide (DMSO). The drugs were finally dissolved in ACSF, and bath applied directly onto the cerebellar surface by a peristaltic pump (Gilson Minipulse 3; Villiers, Le Bel, France) at 0.5 ml/min. For blocking receptors during induction of MLI-PC plasticity, all chemicals were routinely contained in ACSF throughout the experiments. For experiments of PKC inhibition and Ca^2+^ depletion, chelerythrine or CPA were applied at least 30 min before the cell-attached recordings were performed. The concentrations of CRF, BMS-763,534, antisauvagine-30, AM251, chelerythrine and CPA were determined based on previous reports [11, 12, 14, 28, 41, 42].

Electrophysiological data were analyzed with Clampfit 10.6 (Molecular Devices, Foster City, CA). All the parameters were maintained constant for an individual recorded PC in each experiment. Values are expressed as the mean ± S.E.M. One-way ANOVA followed by Tukey’s post-hoc test (SPSS software; Chicago, IL) was used to determine the level of statistical significance between groups of data. *P*-values below 0.05 were considered to indicate a statistically significant difference between experimental groups.

## Supplementary Information

**Additional file 1.** Effect of CRF on facial stimulation-evoked MLI-PC synaptic transmission. **Fig. S1.** Effect of CRF on facial stimulation-evoked MLI-PC synaptic transmission. (A), Representative cell-attached recording traces showing air-puff stimulation (10 ms, 60 psi; arrows)-evoked responses in a cerebellar PC before (Pre CRF) and after (Post CRF) application of CRF (100 nM). (B) Summary of data showing the time course of normalized P1 amplitude before and after application of CRF. (C, D) Bar graphs showing the normalized amplitude of P1 (C) and the normalized pause of simple spike firing (D) before (Pre CRF), after (Post CRF) administration of CRF. n = 6 mice in each group.

**Additional file 2.** Identification of facial stimulation-induced cerebellar MLI-PC synaptic transmission. **Fig. S2.** Facial stimulation induced cerebellar MLI-PC GABAergic synaptic transmission * in Vivo* in mice. (A) Representative cell-attached recording traces showing air-puff stimulation (10 ms, 60 psi; arrows) of ipsilateral whisker pad-evoked responses in a cerebellar PC in treatment with ACSF, GABAzine (20 μM) and recovery (washout). (B) Bar graph with individual data showing the normalized amplitude of P1 in treatment with ACSF, GABAzine and recovery. (C) Mean (± S.E.M.) with individual data showing the normalized pause of simple spike in treatment with ACSF, GABAzine and recovery. n = 8 mice in each group.

## Data Availability

All data generated or analysed during this study are included in this published article and its supplementary information files.
