# Supplementary material for: Opposing actions of CRF-R1 and CB1 receptor on facial stimulation-induced MLI-PC plasticity in mouse cerebellar cortex
Source: BMC Neurosci. 2022 Jun 26;23:39. doi: 10.1186/s12868-022-00726-8 (PMC9235104; doi:10.1186/s12868-022-00726-8)
Supplement: Supplementary file 1 — Additional file 1. Effect of CRF on facial stimulation-evoked MLI-PC synaptic transmission. Fig. S1. Effect of CRF on facial stimulation-evoked MLI-PC synaptic transmission. (A), Representative cell-attached recording traces showing air-puff stimulation (10 ms, 60 psi; arrows)-evoked responses in a cerebellar PC before (Pre CRF) and after (Post CRF) application of CRF (100 nM). (B) Summary of data showing the time course of normalized P1 amplitude before and after application of CRF. (C, D) Bar graphs showing the normalized amplitude of P1 (C) and the normalized pause of simple spike firing (D) before (Pre CRF), after (Post CRF) administration of CRF. n = 6 mice in each group. [file 12868_2022_726_MOESM1_ESM.pdf]

Effect of CRF on facial stimulation-evoked MLI-PC synaptic transmission

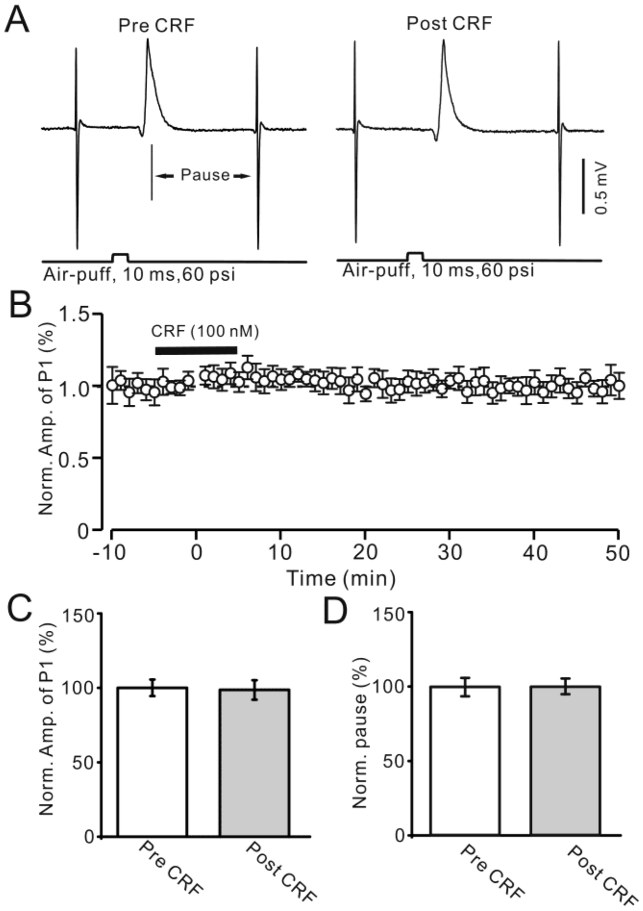

Fig. S1. Effect of CRF on facial stimulation-evoked MLI-PC synaptic transmission. (A), Representative cell-attached recording traces showing air-puff stimulation (10 ms, 60 psi; arrows)-evoked responses in a cerebellar PC before (Pre CRF) and after (Post CRF) application of CRF (100 nM). (B) Summary of data showing the time course of normalized P1 amplitude before and after application of CRF. (C, D) Bar graphs showing the normalized amplitude of P1 (C) and the normalized pause of simple spike firing (D) before (Pre CRF), after (Post CRF) administration of CRF. n = 6 mice in each group.
