# Supplementary material for: Opposing actions of CRF-R1 and CB1 receptor on facial stimulation-induced MLI-PC plasticity in mouse cerebellar cortex
Source: BMC Neurosci. 2022 Jun 26;23:39. doi: 10.1186/s12868-022-00726-8 (PMC9235104; doi:10.1186/s12868-022-00726-8)
Supplement: Supplementary file 2 — Additional file 2. Identification of facial stimulation-induced cerebellar MLI-PC synaptic transmission. Fig. S2. Facial stimulation induced cerebellar MLI-PC GABAergic synaptic transmission in Vivo in mice. (A) Representative cell-attached recording traces showing air-puff stimulation (10 ms, 60 psi; arrows) of ipsilateral whisker pad-evoked responses in a cerebellar PC in treatment with ACSF, GABAzine (20 μM) and recovery (washout). (B) Bar graph with individual data showing the normalized amplitude of P1 in treatment with ACSF, GABAzine and recovery. (C) Mean (± S.E.M.) with individual data showing the normalized pause of simple spike in treatment with ACSF, GABAzine and recovery. n = 8 mice in each group. [file 12868_2022_726_MOESM2_ESM.pdf]

Identification of facial stimulation-induced cerebellar MLI-PC synaptic transmission in vivo in mice

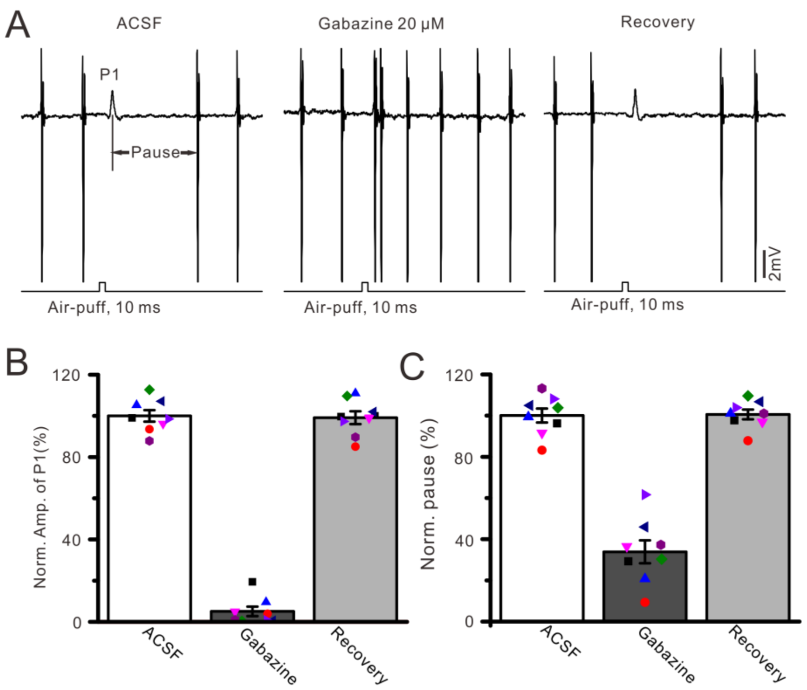

Fig. S2. Facial stimulation induced cerebellar MLI-PC GABAergic synaptic transmission in vivo in mice. (A) Representative cell-attached recording traces showing air-puff stimulation (10 ms, 60 psi; arrows) of ipsilateral whisker pad-evoked responses in a cerebellar PC in treatment with ACSF, GABAzine (20 μM) and recovery (washout). (B) Bar graph with individual data showing the normalized amplitude of P1 in treatment with ACSF, GABAzine and recovery. (C) Mean (± S.E.M.) with individual data showing the normalized pause of simple spike in treatment with ACSF, GABAzine and recovery. n = 8 mice in each group.
